# Individualized medicine: Sex, hormones, genetics, and adverse drug reactions

**DOI:** 10.1002/prp2.541

**Published:** 2019-12-06

**Authors:** Ann M. Moyer, Eric T. Matey, Virginia M. Miller

**Affiliations:** ^1^ Laboratory Medicine and Pathology Mayo Clinic Rochester MN USA; ^2^ Medical Therapy Management and Center for Individualized Medicine Mayo Clinic Rochester MN USA; ^3^ Departments of Surgery, and Physiology and Biomedical Engineering Women's Health Research Center Mayo Clinic Rochester MN USA

**Keywords:** estrogen, men, testosterone, transgender, women

## Abstract

Clinically relevant adverse drug reactions differ between men and women. The underlying physiological and pharmacological processes contributing to these differences are infrequently studied or reported. As gene expression, cellular regulatory pathways, and integrated physiological functions differ between females and males, aggregating data from combined groups of men and women obscures the ability to detect these differences. This paper summarizes how genetic sex, that is, the presence of sex chromosomes XY for male or XX for female, and the influence of sex hormones affect transporters, receptors, and enzymes involved in drug metabolism. Changing levels of sex steroids throughout life, including increases at puberty, changes with pregnancy, and decreases with age, may directly and indirectly affect drug absorption, distribution, metabolism, and elimination. The direct and indirect effects of sex steroids in the form of exogenous hormones such as those used in hormonal contraceptives, menopausal hormone treatments, transgender therapy, and over‐the‐counter performance enhancing drugs may interfere with metabolism of other pharmaceuticals, and these interactions may vary by dose, formulation, and mode of delivery (oral, injection, or transdermal) of the steroid hormones. Few drugs have sex‐specific labeling or dosing recommendations. Furthermore, there is limited literature evaluating how the circulating levels of sex steroids impact drug efficacy or adverse reactions. Such research is needed in order to improve the understanding of the impact of sex hormones on pharmacological therapies, particularly as medicine moves toward individualizing treatments.

**Abbreviations**
ADR: adverse drug reactions
CAR: constitutive androstane receptor
CYP: cytochrome P enzymes
FDA: Food and Drug Administration
GPR30: G‐coupled receptors
ILG1R: insulin‐like growth factor 1 receptor
PI3K: phosphatidyl inositol 3,4,5, triphosphate
PXR: pregnane X receptor
SRY: sex determining region Y
SSRIs: selective serotonin reuptake inhibitors

## 1 INTRODUCTION

Adverse drug reactions are common and thought to be the fourth leading cause of death.^1^ Pharmacogenomic approaches to individualizing pharmacologic therapy involve genetic testing to predict both medication response and adverse reactions to therapy. For individuals predicted to be at higher risk for adverse drug reactions (ADR), a different medication or dose can be prescribed. ADR can range in severity from minor discomfort to death. Depending on the impact on quality of life, ADR can lead to poor medication adherence or discontinuation of therapy. Studies now recognize that there are sex differences in ADRs.^2^ Historically, women were not included in clinical trials because investigators thought women were difficult to study as a result of fluctuating hormone levels throughout the menstrual cycle. In addition, fears of experimental medications being teratogenic to a potential developing fetus resulted in the United States Food and Drug Administration (FDA) excluding women from phase 1 and 2 clinical trials in 1977. As result, ADR disproportionately affecting women may not have been recognized until after medications were approved and on the market, as is often the case with rare genetic differences that can place individuals at risk for rare adverse events not captured during clinical phase trials. In 1993, a law was passed in the United States requiring clinical studies funded by the National Institute of Health to include women. As of 2009, an analysis found that most studies had an average enrollment of only 37% women and the majority (64%) did not stratify results by sex, potentially obscuring differences in efficacy or adverse event outcomes between men and women.^3^

**Figure 1:**
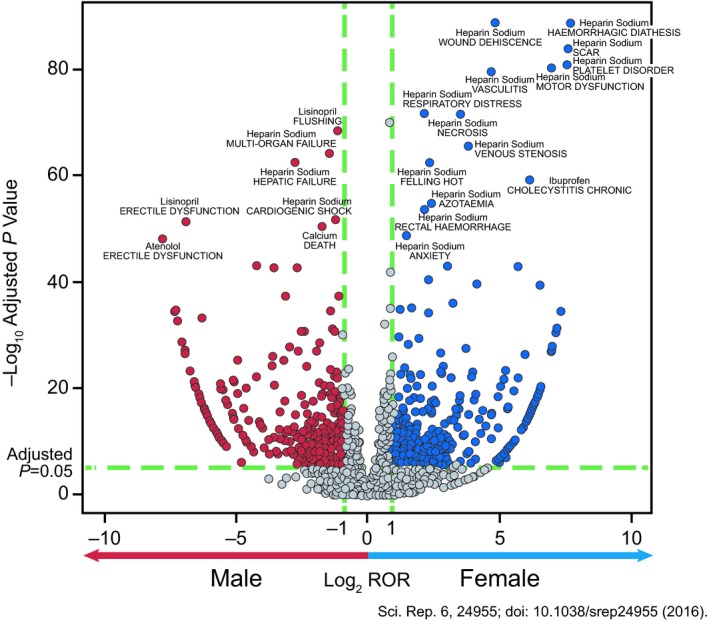
Volcano plot of adverse drug event signals. In the volcano plot of ADE signals, the signal detection result shows the magnitude (log2 reporting odds ratio [ROR], x‐axis) and significance (−log10 adjusted *P* value, y‐axis) for sex‐drug‐event combinations associations of specific drugs. Each spot represents a specific drug‐drug‐event combination interaction. The dashed horizontal green line signals statistical significance threshold (*P* ≤ .05 after adjustment with Bonferroni correction). Two vertical green lines show the threshold of ROR (log2 ROR > 1 or < −1). The blue spots represent the drug‐event combinations more frequently associated with female patients; the red spots, drug‐event combinations more frequently associated with male patients. Reprinted with permission from Reference [2]

In spite of problems with data reporting by sex, over the past several decades, studies have recognized that ADR are experienced by women more often than men 4, 5, 6, 7 with a 1.6‐fold higher odds ratio (Figure 1). This topic is garnering more attention and additional studies. A 2018 study performed in the Netherlands evaluating ADR related to the use of selective serotonin reuptake inhibitors (SSRIs) from 2003 to 2016 found that among the 6791 ADR reports, 68% involved women; however, the percentage of severe reactions was higher among men (31.6% *vs* 22.9%). Most ADR that impacted women were dose‐related and were common reactions mentioned in the product labeling. Among 59 ADR that were reported at least 50 times, 16 were reported more often in women than in men and four were more commonly reported in men than in women.^8^ In the United States, a 2016 study used the US Food and Drug Administration Adverse Event Reporting System to evaluate sex differences in ADR across a wide range of treatments. This study, which included the top 20 long‐term treatment regimens in the US as well as 668 specific drugs, found significant sex differences in ADR for 307 of those medications. Some of these differences could be attributed to medications that are typically only used in one sex or resulted in sex‐specific adverse events (eg prostate cancer). After removing these, 266 sex differences in medications remained.^2^ A similar large‐scale study involving ADRs reported to the pharmacovigilance center Lareb in the Netherlands accounted for gender differences in the number of medication users, which is important given that women use more and different medications than men. A “possibly relevant” sex difference was identified in 15% of the drug‐ADR combinations after accounting for differences in medication use; the risk was higher for women than men in 89% of cases.^9^ If personalized medicine is to become a reality, it is critical to understand not only interindividual genetic differences, but also underlying physiological differences between males and females that additionally contribute to sex differences in ADR. Recognition and understanding of sex differences may lead to further improvements in outcomes when combined with traditional and pharmacogenomic approaches to medication selection as a part of shared decision‐making between the patient and physician.

## 2 BASIC MECHANISMS

### 2.1 Physiologic differences

The presence of sex chromosomes, XX in female and XY in males, in all nucleated cells represents the most fundamental genetic difference between females and males. Genes on these chromosomes regulate specific and diverse physiological functions, in part, through modulation of genes on the autosomes.^10^, ^11^ In addition, the *SRY* gene on the Y chromosome encodes the sex‐determining region Y (SRY) protein, which is a DNA‐binding protein that is important in the development of the testes that will ultimately secrete testosterone and lead to a male phenotype. The *AR* gene that encodes the androgen receptor resides on the X chromosome; therefore, any variant in *AR* that impacts function will be expressed as an X‐linked trait in males with one X chromosome.^12^, ^13^, ^14^ In females, variants in genes on the X chromosome that undergo X inactivation typically show mosaic expression of the phenotype in a particular tissue or system, or may lead to skewed lyonization.^15^, ^16^, ^17^, ^18^

As the reproductive organs form, sex steroid hormones differing between males and females will be secreted and influence body size, organ size, tissue composition (lean body mass, body fat), gastric acid secretion, gastric emptying time, circulating proteins that may bind medications, renal function, immune reactivity, and drug metabolizing enzymes;^19^ all of which influence absorption, distribution, metabolism, and elimination of drugs. While many of these differences may influence medication efficacy and toxicity can be attributed, in part, to underlying differences in hormone expression, hormonal status and potential interactions of pharmaceuticals with pathways modulated by sex steroids are usually not considered in preclinical studies for drug development, clinical trials, or reporting of ADRs.

### 2.2 Sex steroid signaling mechanisms

**Figure 2:**
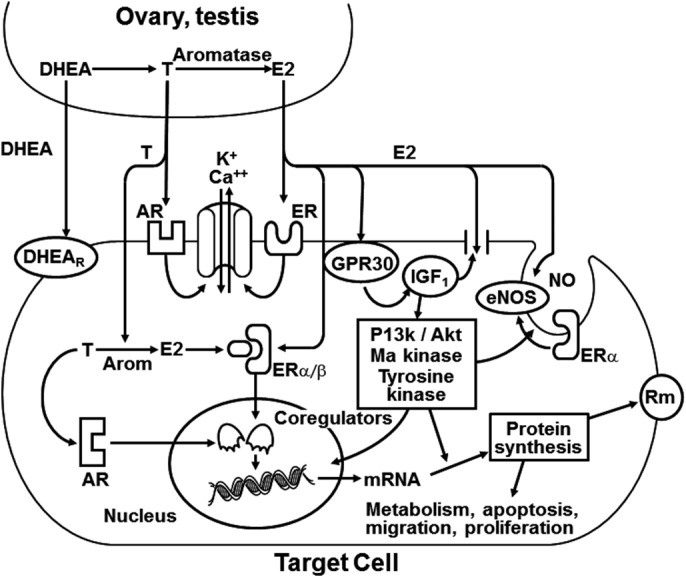
Schematic representation of potential cellular mechanisms by which sex steroids influence rapid nongenomic signaling and modulation of gene transcription (ie, genomic effects). Signaling can occur through surface receptor modulation of ion channels and transporters. At the nuclear levels, hormone‐bound receptors may compete for the same nuclear coregulators. Taken together, acute and sustained treatment of cells with sex steroid hormones have the potential to influence all aspects of cellular function directly through activation of ion channels, transporters and enzymes, and indirectly through genomic regulation for expression of enzymes, structural proteins, and membrane receptors. AR, androgen receptor; Arom, aromatase; DHEA, dehydroepiandrosterone; E2; 17β estradiol; ER, estrogen receptor; eNOS; endothelial nitric oxide synthase; GPR30, G protein‐coupled estrogen receptor; IGF_1_, insulin‐like growth factor 1; mRNA, messenger ribonucleic acid; NO, nitric oxide; Rm, membrane receptor

Traditionally, sex hormone signaling mechanisms were only thought to alter gene transcription through steroid response elements on specific genes, that is, genomic effects (Figure 2).^20^ However, these mechanisms did not account for the rapid changes in cellular functions when steroids were applied acutely in experimental settings.^21^, ^22^ Although some of the rapid effects observed could be attributed to antioxidant properties of the steroids, surface G‐coupled receptors (GPR30) that are independent of estrogen receptors but responsive to estrogen were identified as important in nongenomic estrogen signaling mechanisms.^23^, ^24^, ^25^ Estrogen receptors are ubiquitous with distribution in many cell types including hepatocytes.^26^, ^27^, ^28^ Activation of the GPR30 receptor and interaction with insulin‐like growth factor 1 receptor (ILG1R) initiates a cascade of activation of phosphatidyl inositol 3,4,5, triphosphate (PI3K)/Akt (serine‐threonine kinase) and mitogen‐activated protein kinases^24^, ^25^, ^29^ that has numerous intracellular effects, including promoting expression of Bc1‐2. Bc1‐2 affects apoptosis,^26^ endocytosis of canalicular transporters such as multidrug resistance‐associated protein 2 (Abcc2) and bile salt export pump (Abcb11),^28^ and synthesis of high‐density lipoprotein cholesterol and liver fat in female but not male rats.^27^ These same estrogen signaling pathways, if present in humans, may impact pharmacodynamic and/or pharmacokinetic pathways by altering similar physiologic processes, such as endocytosis of drug transporters. These interactions warrant further study.

### 2.3 Sex steroids and pharmacokinetic interactions with medications

**Figure 3:**
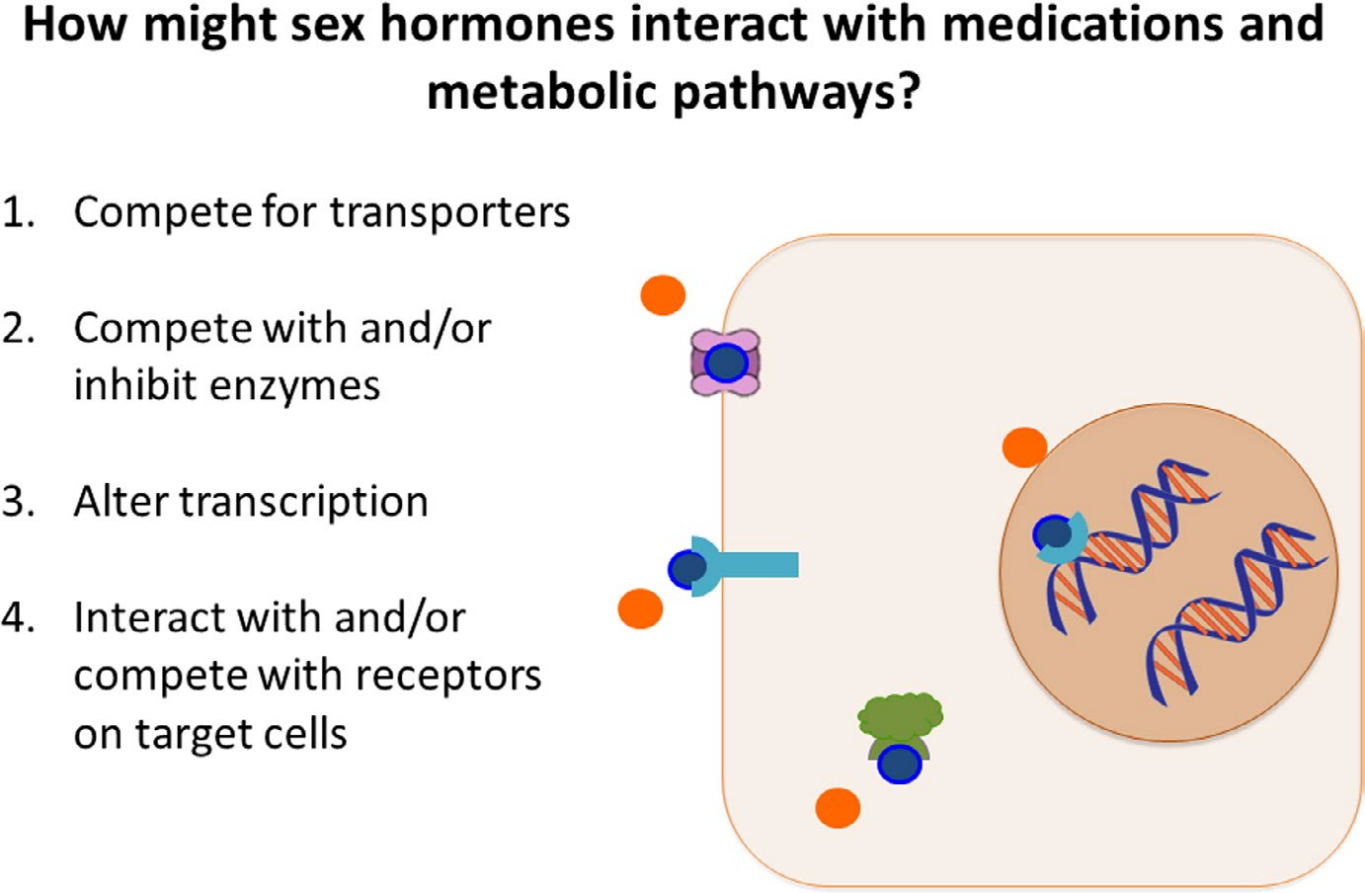
Schematic representation of potential mechanisms through which sex steroid hormones can affect drug metabolism and actions leading to adverse drug reactions. The orange circles represent a medication and the blue circles represent a hormone that is competing with the medication. The purple symbol represents a transporter. The green symbol represents an enzyme. The blue symbol on the cell surface represents a receptor. The blue symbol interacting with the DNA represents a transcription factor

There are several mechanisms by which sex hormones may interact with medications and their metabolic pathways, contributing to ADR (Figure 3).

#### 2.3.1 Absorption

Bioavailability of a drug may depend on mode of delivery and formulation. For example, transdermal formulations often require absorption into subcutaneous adipose tissue. Women typically have more subcutaneous adipose tissue than men, which theoretically could impact absorption.^19^ In contrast, orally administered medications are absorbed through the gastrointestinal tract where sex differences in gastric pH, gastric emptying time, intestinal transit time, first‐pass metabolism, and other variables may impact absorption. For example, when verapamil is orally administered, it is cleared faster by men than women; however, this is not the case when the medication is administered intravenously.^30^ Orally administered drugs often undergo hepatic first‐pass metabolism, where there may be sex differences in enzyme expression, while medication administered intravenously or absorbed through the skin or other mucus membranes (nasal, vaginal) will reach the circulation without first past metabolism. Due to sex differences in these variables, different routes of administration may theoretically be more effective and/or less toxic for individuals of one sex; however, currently limited data exist to support or refute this idea.

#### 2.3.2 Competition for transporters

Drugs may compete for transporters into cells, thus affecting downstream metabolism or availability at the drug target and altering extracellular concentrations. For example, the OATP1B1 transporter, encoded by the gene *SLCO1B1*, is responsible for transport of estrogens including estrone‐3‐sulfate and estradiol 17β‐D‐glucuronide. Statin drugs are also transported by OATP1B1. Competitive inhibition of this transporter may occur when multiple substrates are present.^31^ Several studies have found sex‐specific effects of *SLCO1B1* genetic variants on the efficacy of statin treatment.^32^, ^33^ Increased risk of statin‐related myopathy is associated with female sex, particularly among carriers of the *SLCO1B1* c.521C allele, suggesting that competition for transporters may result in clinically significant drug‐hormone interactions.^34^ In addition, there may be sex differences in transporter expression.^35^

#### 2.3.3 Competition and/or regulation of expression of drug metabolizing enzymes

The pregnane X receptor (PXR) and constitutive androstane receptor (CAR) regulate expression of cytochrome P450s, including *CYP3A4*, and other genes, and these receptors are activated by a variety of compounds, including steroid hormones.^36^ Hepatic CYP3A4 activity is known to be higher among women than men,^37^ although sex differences in the oral clearance of CYP3A4 substrates have not been consistently reported.^38^ Changes in enzyme expression associated with hormones will be described in more detail in the sections on pregnancy and hormonal contraception below.

#### 2.3.4 Sex steroids and pharmacodynamic interactions with medications

Steroid sex hormone status may also influence pathways contributing to the mechanism of action of medications; however, less is known related to these pharmacodynamic interactions. In one example, women were found to require a smaller dose of olanzapine in order to achieve 70% occupancy of the dopamine D2 receptor for medication efficacy.^39^ Testosterone and/or estrogen may modulate the pharmacodynamics of olanzapine at the D2 receptors as adjusting for weight, height, age, or concomitantly administered medications did not affect olanzapine clearance.^40^ In addition, review of anti‐obesity drugs suggests that pharmacokinetic, pharmacodynamic, and nonpharmacologic sex differences may influence success in reaching weight loss goals.^41^

## 3 FEMALE‐SPECIFIC CONSIDERATIONS: INFLUENCES OF ENDOGENOUS AND EXOGENOUS SEX STEROIDS HORMONES

Fluctuations in endogenous sex steroid hormones that occur naturally with the menstrual cycle, pregnancy, and with the transition to menopause have the potential to influence drug efficacy and ADRs.^42^ Additionally, women use exogenous hormones as a medication for contraceptives, treatments for hot flashes, night sweats, vaginal dryness, and a variety of other indications. Thus, these hormonal treatments could be considered both as medication and a source of ADRs, as well as modifiers of other drug actions. That is, exogenous hormones may affect other medications by altering metabolism, while at the same time drug metabolism pathways may impact exogenous hormones used for therapy. Interindividual variation in components of metabolic pathways (ie, pharmacogenetics) may lead to differences in response to exogenous hormones. With further research, it may be possible one day to use a pharmacogenetic approach, tailoring hormone therapy regimens to the individual.

### 3.1 Pregnancy

Changes in endogenous hormones associated with pregnancy will influence drug efficacy and some medications may have adverse effects on fetal development. Indeed, developmental defects associated with use of thalidomide resulted in the exclusion of pregnant women from drug testing trials.^43^ However about 64% of pregnant women take a medication (other than vitamin supplements), 2/3 of which may not have been tested in pregnant women.^44^, ^45^

Pregnancy does not simply increase the volume of blood and extracellular fluid that influence distribution and clearance of drugs, but the hormonal changes also influence enzyme activity. For example, activity of CYP1A2 is decreased during pregnancy affecting metabolism of caffeine and theophylline.^46^ In addition, the CYP2C19 enzyme may be inhibited by endogenous sex steroids during pregnancy.^46^ On the contrary, activity of other enzymes increase, primarily through the second and third trimesters, including CYP2C9, CYP3A4, and UGT1A4. Activity of other enzymes, such as CYP2D6, vary throughout the pregnancy and may differ by trimester.^47^ The effect of hormonal changes with pregnancy on drug transporter genes is not well understood, but may involve activation of estrogen and androgen receptors.^48^

### 3.2 Menopause

With menopause, circulating estrogen decreases to about 90%. Conversion of androgens to estrogen by aromatase (encoded by *CYP19A1*) in adipose tissue and skin becomes the predominant source of estrogen. With the decrease in estrogen, changes in other drug metabolizing enzymes are also observed. For example, CYP3A4 activity in the intestine is reduced by about 20%,^49^ thus, medications utilizing this pathway would be impacted over the menopause transition and aging past menopause.

Exogenous hormones used to treat symptoms of menopause (hot flashes, night sweats, vaginal dryness, sleep disturbances) may affect medications by altering metabolism; at the same time, drug metabolism pathways may impact exogenous hormones used for therapy. The genes encoding drug metabolizing enzymes and transporters are highly polymorphic, which is the basis of pharmacogenomics. Although the influence of genetic variation on menopausal physiology is not well understood, heritability estimates of age at menopause ranges from 31%‐78%. Thus, the question arises as to whether a pharmacogenomic approach, that is, studying the impact on exogenous hormones of genetic variation in genes encoding pathways of drug metabolism, could be applied to optimize menopausal hormone therapy or hormone therapy for other indications. This question is especially important to optimize hormone therapy for women who might undergo bilateral oophorectomy prior to the age of natural menopause and require menopausal hormone therapy to reduce the risk of multimorbidity of aging.^50^, ^51^, ^52^

Our group has taken a candidate gene approach to begin to evaluate how genetic variants in enzymes involved in estrogen metabolism might be related with response to estrogen therapies. One variant in *SULT1A1*, which encodes an enzyme that sulfates estrone, 17β‐estradiol, and 4‐methoxy‐estradiol might be associated with serum hormone levels of estrogen and menopausal symptoms. The *SULT1A1* gene is polymorphic with both single nucleotide variants (SNVs) and whole gene deletions and duplications (copy number variation, CNV). When evaluating this gene, it is critical to consider the impact of both SNV and CNV simultaneously. Some of the SNVs decrease enzyme activity, while others impact transcription; gene duplication also leads to increased activity.^53^, ^54^ In the Kronos Early Estrogen Prevention Study (KEEPS) of recently menopausal women randomized to either placebo, oral conjugated equine estrogen, or transdermal 17β‐estradiol, women with increased number of G alleles at rs9282861, which would be expected to increase SULT1A1 activity, experienced menopause at a younger age, and reported less severe night sweats but increased frequency of insomnia at baseline prior to the initiation of the hormone treatment.^54^ When randomized to active drug, there was no significant relationship between the genotype and menopausal symptoms. However, in women randomized to oral conjugated equine estrogen, variants in *SULT1A1* affected circulating levels of sulfated estrone and the ratio of sulfated estrogen to estrogen.^54^

A second candidate gene *SLCO1B1* encodes the transporter (OATP1B1) for many endogenous and xenobiotic compounds. In the context of this paper, this transporter transports estradiol‐17β‐glucuronide, estrone‐3‐sulfate, and statins from the blood into hepatocytes. Variants in *SLCO1B1* associated with breast cancer risk and statin‐induced myopathy in postmenopausal women using oral conjugated equine estrogen with medroxyprogesterone acetate. In KEEPS, women with normal transporter activity had lower circulating levels of estrone and 17β‐estradiol sulfate than those with lower activity, while those with reduced transporter activity demonstrated a greater decrease in night sweats among women on active treatment.^55^ Additional research using larger cohorts of women is needed in order to utilized a pharmacogenomic approach to maximize hormone treatments. Research using a pharmacogenomic approach is also needed to examine declines in testosterone (andropause/androdrift) in age‐matched men.

#### 3.2.1 Hormonal contraception

Exogenous hormones may be prescribed for a variety of purposes, including contraception. These exogenous hormones also impact metabolism of other medications. While the underlying mechanism of decreased CYP activity in the presence of exogenous hormones, such as those in oral contraceptives, is thought to be a competitive inhibition, there is some evidence that estradiol may downregulate CYP2C19 expression through the interaction of estrogen receptor (ER) α with a binding site in the *CYP2C19* promoter.^48^ Oral contraceptives are examples of exogenous hormones that modulate drug metabolism through inhibition of multiple cytochrome P450 enzymes including CYP1A2, CYP3A4, CYP2C19, and CYP2C9‐mediated metabolism.^56^, ^57^, ^58^, ^59^

Genetic variation may also influence the concentrations of the exogenous hormones used for contraception and other indications. A study of 350 healthy, reproductive‐age women using etonogestrel implants for 12‐36 months were genotyped for 14 genes encoding proteins involved in steroid hormone‐related pathways.^60^ Carriers of the CYP3A7*1C allele were more likely to have serum etonogestrel concentrations falling below the threshold of 90 pg/mL required for consistent ovulatory suppression. This allele results in adult expression of the fetal CYP3A7 enzyme, which is in the same family as the CYP3A4 enzyme known to metabolize estrogens. In addition, nongenetic variables of body mass index and duration of implant use also impacted estrogen levels. This example further highlights the importance of understanding individual variation in steroid hormone metabolism in ensuring efficacy of therapy.

## 4 TRANSGENDER

**Figure 4:**
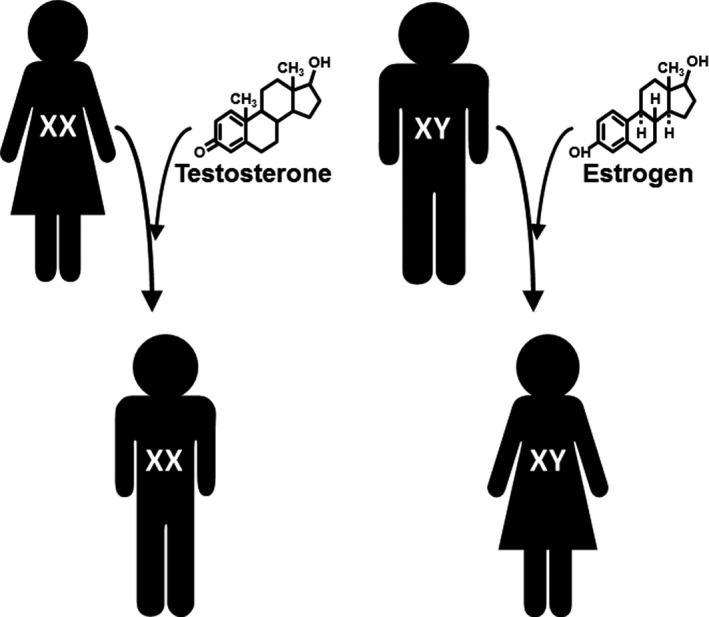
Depiction of activational effects of sex steroids in individuals undergoing treatment for gender incongruence. Endogenous production of hormones defined by the presence of sex chromosomes are suppressed (or the gonads removed) with the subsequent administration of hormones from the nonbiological gonads. Thus, the genetic sex of the individual remains the same and the exogenous hormones can be considered as a medication that carry pharmacological and physiological risks with the potential to interact and modify efficacy of other medications given for a nonreproductive condition, that is, depression, hypercholesterolemia, arthritis, cancer, etc.

Changes in outward appearance, physiology, and metabolism resulting from treatment with sex steroid hormones for clinically defined gender incongruence^61^ reflect activational effects of the hormones. However, these activational effects are expressed on the background of the sex chromosomes present at birth (Figure 4). Prescription guidelines for dosing, mode of delivery, and timing of this type of therapy have evolved based, in part, on reports of adverse events associated with certain formulations.^62^ For trans men, (XX with testosterone treatment), general physiological and metabolic effects include increases in body mass index, modest increases in blood pressure, and increases in serum triglycerides and low‐density lipoprotein cholesterol.^63^, ^64^ Adverse cardiovascular events include increased risk for venous thromboembolism, but this has been attributed to an oral formulation of testosterone,^61^ and in some cases increased risk of myocardial infarction.^63^, ^65^ For trans women, in addition to risks for venous thromboembolism associated with oral formulations of the steroids, there is also increased risk for stroke and myocardial infarction.^65^, ^66^ A challenge in evaluating risks and adverse outcomes associated with transgender therapy is accounting for the appropriate reference population, for example, adverse events in trans men compared to cis women or cis men and vice versa for trans women.^66^ This challenge is also confounded by differences in formulations of hormone therapy, age at initiation of therapy, and comorbid conditions that, by themselves, may present as risk factors for adverse cardiovascular events. To date, there are no published longitudinal studies that have included data on trans persons prior to and serially after treatment into older age, however, the Gender Dysphoria Treatment in Sweden (GETS) study is designed to do so.^67^

Few studies have specifically addressed potential hormone‐drug interactions except for managing trans patients with sickle cell disease,^68^ and treatments for HIV.^69^ Although both trans men and trans women report reductions in anxiety, depression and perceived stress when treated with hormone therapy,^61^ individuals using pharmaceutical treatments for other conditions prior to initiation of the steroid hormones may require adjustment for those medications. This adjustment may be due to the drug‐hormone interactions involving metabolic pathways discussed previously or due to interactions of steroids hormones with molecular targets for those drugs.^70^ Hormone‐drug interactions for other medications, statins, for example, are unknown in the trans population given the age of the individuals when therapy was initiated and the impact of the hormones on metabolic factors that influence development of atherosclerosis with aging in cis men. These considerations need to be addressed in future studies.

## 5 SUMMARY

Although there are data supporting sex differences in ADRs, there are few systematic analyses of these adverse events by drug class that take into account stages of life (age), and hormonal status. Given that sex hormones may alter the pharmacokinetics and pharmacodynamics of medications, it is important to note that these hormones naturally fluctuate throughout the lifespan. Among females, hormones change at puberty, with menstrual cycles, during pregnancy, and at menopause; in addition, exogenous hormones may be used for contraception or to alleviate menopausal symptoms. Among males, hormones change at puberty as well as with aging, and exogenous hormones may be used. However, many preclinical basic pharmacology studies to define mechanisms of disease (with a potential to identify a new drug target) do not identify the sex of the experimental material, age or hormonal status of the source of cells or tissues.^71^, ^72^, ^73^ While males are disproportionately included in studies, the impact of aging (which influences sex steroid hormone levels) and exogenous hormone use are not necessarily specifically studied. Large databases exist that should allow discernment of types of adverse reactions between men and women but these should consider age, hormonal status, specific medication/probe drug used, and interindividual variation in enzyme activity due to genetic variation. In addition, analysis of large databases is often limited by lack of other information that might affect the outcome including interactions with over‐the‐counter medications, environmental exposures, and cultural or psychosocial influences that affect compliance.^74^ Even with these limitations, newer clinical studies need to consider reporting outcomes by sex, age, and hormonal status even if the study is not powered to determine a statistical difference. Understanding clinical parameters together with genetic information for complex disease and drug response phenotypes within the context of biological sex and hormonal status is critical for individualized medicine.

## DISCLOSURE

The authors declare no financial conflicts of interest. VMM research is supported by a grant from the National Institutes of Health U54 AG 44170.

## AUTHOR CONTRIBUTIONS

AMM: Drafted and edited the manuscript and figures. ETM: Drafted and edited the manuscript. VMM: Drafted and edited the manuscript and figures.

## DATA RESPOSITORY LINK

Not applicable as only previously reported data are presented in this review.

## ETHIC STATEMENT

All published studies involving humans contain a statement that the work was performed in accordance with review by Internal Review Boards and the Declaration of Helsinki and that participants signed consent documents. Papers reporting work conducted on experimental animals were conducted in accordance with the Animal Welfare Act.
